# Clustering of eyes with age-related macular degeneration or pachychoroid spectrum diseases based on choroidal thickness profile

**DOI:** 10.1038/s41598-021-84650-7

**Published:** 2021-03-02

**Authors:** Young Ho Kim, Boram Lee, Edward Kang, Jaeryung Oh

**Affiliations:** Department of Ophthalmology, Korea University College of Medicine, 73 Goryeodae-ro Sungbuk-ku, Seoul, 02841 Korea

**Keywords:** Biomarkers, Diseases, Medical research, Pathogenesis

## Abstract

Choroidal changes have been suggested to be involved in the pathophysiology of both age-related macular degeneration (AMD) and pachychoroid spectrum diseases (PSD). To find out the choroidal characteristics of each disease groups, various groups of AMD and PSD were classified into several clusters according to choroidal profiles based on subfoveal choroidal thickness (CT), peripapillary CT, the ratio of subfoveal CT to peripapillary CT and age. We retrospectively analyzed 661 eyes, including 190 normal controls and 471 with AMD or PSDs. In the AMD groups, eyes with soft drusen or reticular pseudodrusen were belonged to the same cluster as those with classic exudative AMD (all *p* < 0.001). However, eyes with pachydrusen were not clustered with eyes from other AMD groups; instead, they were classified in the same cluster as eyes from the PSD group (all *p* < 0.001). In the PSD group, eyes with pachychoroid neovasculopathy were grouped in the same cluster of those with polypoidal choroidal vasculopathy (*p* < 0.001). The cluster analysis based on the CT profiles, including subfoveal CT, peripapillary CT, and their ratio, revealed a clustering pattern of eyes with AMD and PSDs. These findings support the suggestion that pachydrusen has the common pathogenesis as PSD.

## Introduction

Typical age-related macular degeneration (AMD), which is characterized by the development of drusen, is a chronic degenerative disease of the photoreceptors, retinal pigment epithelium (RPE), Bruch’s membrane, and the choriocapillary in the human macula^1,2^. Vision can be preserved in the early stage of the disease in eyes with drusen. However, in the late stage, visual prognosis is poor due to the development of center-involving geographic atrophy (GA) or exudative maculopathy from macular neovascularization (MNV). In recent studies, various forms of exudative maculopathy have been presented in old age^3^. In addition to pachychoroid neovasculopathy (PNV), polypoidal choroidal vasculopathy (PCV) was presented as a main cause of exudative maculopathy in Asians^4–7^. Additionally, several diseases that share the characteristic of choroidal vessel engorgement or thickening^3–6^, are classified into pachychoroid spectrum diseases (PSDs). However, it is unclear whether the PCV and PNV are variants of AMD or a distinct disease entity^8^, and it is often difficult to differentiate PCV and PNV from typical neovascular AMD^9^.

The choroid is a vascular coat that supports the retina to maintain stable blood flow, nutrition, and temperature, and enters the back of the eye from the posterior and anterior ciliary artery^10,11^. In many studies, both AMD and PSDs, which occur in the macula and are characterized by drusen or pachyvessel^12^, have been suggested to be associated with choroidal changes in their pathophysiology^2,3,5,12^. With the development of the enhanced depth imaging technique and swept-source optical coherence tomography (SS-OCT), the choroidal thickness (CT) can be measured in vivo, making it possible to measure macular CT in eyes with AMD and PSDs^13–15^. Previous studies with OCT showed variation of macular CT in eyes with AMD and PSDs^13–15^.

AMD and PSDs are diseases that occur in the macula, but that are not limited to the macula^16–19^. In addition, it has been noted that the macular choroid is highly variable due to physiological or pathological reactions^11,20,21^. It has been suggested that the thickness of the choroid at the extra-macular area, as well as the macular area, reflects the characteristics of the patient's choroid^22,23^. In addition to the macular area, studies on the measurement of CT at the extramacular area have been performed, since the characteristics of the whole body of the patient may also be reflected in the choroid outside of the macula^22,24–26^. In addition, measurement of CT at the macular area, and outside of the macula, allows observation of the relative changes in the choroid of the macular area in macular disease^4,27–29^. Although many studies have shown changes in macular CT in eyes with AMD or PSDs, few studies have focused on changes in CT outside the macular area^4,23,27,29^. In this study, we hypothesized that CT profiles, including subfoveal CT, nasal peripapillary CT, and the ratio of subfoveal CT to peripapillary CT, can be the surrogate variables of the choroids in eyes with AMD and PSDs. And, we classified the eyes based on CT profiles into several clusters and compared the distributions of eyes between clusters with various groups of AMD and PSD.

## Results

A total 661 eyes of 661 subjects were included (Table 1). Mean age was 66.0 ± 13.4 years. Female gender was 327 (49.5%). Among 661 eyes, 190 were normal controls with normal fundus and 471 patients had AMD or PSD.

**Table 1:** General characteristics of included patients.

Variables	N (%)
Age, mean ± SD, years	66.0 ± 13.4
Gender	
Female	327 (49.5%)
Male	334 (50.5%)
Hypertension	
Yes	291 (44.0%)
No	370 (56%)
Diabetes	
Yes	127 (19.2%)
No	534 (80.8%)
Axial length, mean ± SD, mm	23.71 ± 1.10
Classification of eyes	
Normal control	190 (28.7%)
Early AMD with soft drusen or reticular pseudodrusen	58 (8.8%)
Group with pachydrusen	69 (10.4%)
Classic exudative AMD	88 (13.3%)
Pachychoroid pigment epitheliopathy	36 (5.4%)
Pachychoroid neovasculopathy	128 (19.4%)
Polypoidal choroidal vasculopathy	92 (13.9%)

SD, standard deviation; AMD, age-related macular degeneration.

The eyes were grouped into several groups as described in method section and further analyses were performed. Briefly, eyes with various types of drusen were grouped into early AMD group with soft drusen or reticular pseudodrusen and group with pachydrusen. Eyes with MNV were classified as a classic exudative AMD group. Eyes with PSDs were classified into PPE, PNV, and PCV groups.

A total 471 eyes were classified into six groups: early AMD group (58 eyes), pachydrusen group (69 eyes), classic exudative AMD group (88 eyes), PPE group (36 eyes), PNV group (128 eyes), and PCV group (92 eyes). Age and gender were different among groups (all *p* < 0.001) (Table 2). Mean age was greatest in the classic exudative AMD group (77.4 ± 7.4 years).

**Table 2:** Comparison of clinical characteristics and choroidal thickness profiles between different AMD and PSD groups.

Variables	Normal control (n = 190)	Early AMD (n = 58)	Pachydrusen (n = 69)	Classic exudative AMD (n = 88)	PPE (n = 36)	PNV (n = 128)	PCV (n = 92)	*P* value
Age, years	55.8 ± 15.2	75.1 ± 6.1	68.6 ± 8.4	77.4 ± 7.4	57.7 ± 12.6	68.8 ± 9.1	68.2 ± 8.7	< 0.001
Gender, female	111 (58.4%)	43 (74.1%)	37 (53.6%)	55 (62.5%)	13 (36.1%)	41 (32.0%)	27 (29.3%)	< 0.001
Hypertension	62 (32.6%)	36 (62.1%)	29 (42.0%)	40 (45.5%)	12 (33.3%)	66 (51.6%)	46 (50.0%)	0.001
Diabetes	27 (14.2%)	15 (25.9%)	18 (26.1%)	17 (19.3%)	7 (19.4%)	27 (21.1%)	16 (17.4%)	0.293
Subfoveal CT, μm	279.5 ± 99.5	175.1 ± 81.7	289.3 ± 96.7	143.4 ± 74.4	314.8 ± 95.7	265.9 ± 109.6	241.4 ± 88.8	< 0.001
Peripapillary CT, μm	136.3 ± 56.6	105.7 ± 49.6	138.5 ± 65.9	91.6 ± 45.8	165.0 ± 69.3	150.4 ± 72.4	127.4 ± 52.1	< 0.001
Ratio of subfoveal CT to peripapillary CT	2.24 ± 0.85	1.81 ± 0.95	2.38 ± 0.94	1.76 ± 0.87	2.12 ± 0.83	1.91 ± 0.74	2.06 ± 0.85	< 0.001

Data are presented as mean ± standard deviation or number (%).

AMD, age-related macular degeneration; Early AMD, early AMD group with soft drusen or reticular pseudodrusen; PPE, pachychoroid pigment epitheliopathy, PNV, pachychoroid neovasculopathy; PCV, polypoidal choroidal vasculopathy; CT, choroidal thickness.

### Characteristics of choroidal thickness

In 190 eyes of normal controls, the mean subfoveal CT was 279.5 ± 99.5 µm and the peripapillary CT was 136.3 ± 56.6 µm, respectively, and they were correlated with each other (*r* = 0.490, *p* < 0.001). In normal controls, subfoveal and peripapillary CT were correlated with age (*r* = − 0.304, *p* < 0.001; *r* = − 0.353, *p* < 0.001). The ratio of subfoveal CT to peripapillary CT was 2.24 ± 0.85 and was not correlated with age (*r* = 0.117, *p* = 0.108). Subfoveal CT, peripapillary CT, or the ratio of subfoveal CT to peripapillary CT was not different between males and females (*p* = 0.686, *p* = 0.642, *p* = 0.377, respectively).

In 471 eyes of with AMD or PSD, the mean subfoveal CT was 234.2 ± 108.8 µm and the peripapillary CT was 128.8 ± 64.7 µm, respectively, and they were correlated with each other (*r* = 0.671, *p* < 0.001). Subfoveal and peripapillary CT were correlated with age (*r* = − 0.408, *p* < 0.001; *r* = − 0.324, *p* < 0.001). The ratio of subfoveal CT to peripapillary CT was 1.98 ± 0.87. Subfoveal and peripapillary CT of males (253.4 ± 114.2 µm, 137.6 ± 69.2 µm) was greater than that of females (211.6 ± 97.7 µm, 118.4 ± 57.2 µm) (*p* < 0.001, *p* = 0.001, respectively). However, the ratio of subfoveal CT to peripapillary CT was not different between males and females (*p* = 0.551).

### Determination of factors for choroidal thickness profile

When considering the difference between age and gender, the estimated marginal means of subfoveal CT were different between groups. Bonferroni-adjusted post hoc tests showed that the mean subfoveal CT in eyes of AMD groups, except the pachydrusen group, was lower than those in normal controls and PSD groups (Table 3). The mean subfoveal CT of the early AMD group was lower than that of normal controls, pachydrusen group, PPE and PNV (*p* = 0.004, *p* < 0.001, *p* < 0.001, *p* < 0.001) (see Supplementary Table S1). The mean subfoveal CT of the classic exudative AMD was also lower than that of normal controls, as well as in the pachydrusen group, PPE, PNV, and PCV groups (all *p* < 0.001). The subfoveal CT of the pachydrusen group was greater than that verified in the early AMD, classic exudative AMD, and PCV groups (*p* < 0.001, *p* < 0.001, *p* = 0.014). However, it was not different from those in normal controls, PPE, and PNV. The mean subfoveal CT of the PSD groups was not different from normal controls. The mean subfoveal CT of the PSD groups, except PCV, was greater than that of the AMD groups, except pachydrusen group (all *p* < 0.001). The subfoveal CT of PCV was significantly greater than the classic exudative AMD group (*p* < 0.001), but lower than the pachydrusen group (*p* = 0.014).

**Table 3:** Estimated marginal means with 95% confidence intervals.

Variables	Estimated marginal mean (μm)*	SE	95% CI	*P* value
**Subfoveal CT**				< 0.001
Normal control	256.7	7.67	241.6 ~ 271.7	
Early AMD	193.1	14.24	165.1 ~ 221.0	
Pachydrusen	295.7	11.17	273.7 ~ 317.6	
Classic exudative AMD	172.2	10.86	150.8 ~ 193.5	
PPE	295.6	16.26	263.7 ~ 327.5	
PNV	268.1	8.81	250.8 ~ 285.3	
PCV	243.1	10.59	222.3 ~ 263.9	
**Peripapillary CT**				< 0.001
Normal control	121.2	4.77	111.8 ~ 130.6	
Early AMD	123.4	8.87	106.0 ~ 140.8	
Pachydrusen	142.2	6.96	128.6 ~ 155.9	
Classic exudative AMD	107.7	6.76	94.4 ~ 121.0	
PPE	147.6	10.13	127.7 ~ 167.5	
PNV	154.6	5.48	143.8 ~ 165.4	
PCV	128.7	6.60	115.7 ~ 141.6	
**Ratio of subfoveal CT to peripapillary CT**				< 0.001
Normal control	2.30	0.07	2.16 ~ 2.43	
Early AMD	1.66	0.13	1.40 ~ 1.91	
Pachydrusen	2.37	0.10	2.17 ~ 2.57	
Classic exudative AMD	1.74	0.10	1.55 ~ 1.94	
PPE	2.22	0.15	1.93 ~ 2.51	
PNV	1.87	0.08	1.71 ~ 2.03	
PCV	2.07	0.10	1.88 ~ 2.26	

SE, standard error; CI, confidence interval; CT, choroidal thickness; AMD, age-related macular degeneration; Early AMD, early AMD group with soft drusen or reticular pseudodrusen; PPE, pachychoroid pigment epitheliopathy, PNV, pachychoroid neovasculopathy; PCV, polypoidal choroidal vasculopathy.

*Evaluated at covariates appeared in the model: Age = 66.03.

The estimated marginal means of peripapillary CT in eyes of the AMD groups were not different from those of normal controls. The peripapillary CT in eyes of the AMD groups, except classic exudative AMD group, was not different from those in the PSD groups. Peripapillary CT of classic exudative AMD group was significantly lower than those of PPE and PNV (*p* = 0.032, *p* < 0.001, respectively). The peripapillary CT of PSD groups except PNV group were not different from those of normal controls. However, the peripapillary CT of PNV group was significantly greater than those of normal controls (*p* < 0.001). The peripapillary CT of PNV group was significantly greater than those of classic exudative AMD group (*p* < 0.001), while the peripapillary CT of PCV group was not different from those of the classic exudative AMD group (*p* = 0.527).

The estimated marginal means of the ratio of subfoveal CT to peripapillary CT in eyes of the early AMD and classic exudative AMD groups were significantly lower than those in normal controls (*p* = 0.001, *p* = 0.001, respectively), but not different from the PSD groups. However, the ratio of subfoveal CT to peripapillary CT in eyes of the pachydrusen group was not different from normal controls but was greater than those in the AMD groups (all *p* < 0.001). The ratio of subfoveal CT to peripapillary CT in eyes of the PSD groups, except the PNV group, was not different from those of normal controls and AMD groups. The ratio of subfoveal CT to peripapillary CT in eyes of the PNV group was greater than those of normal controls (*p* = 0.002).

**Figure 1:**
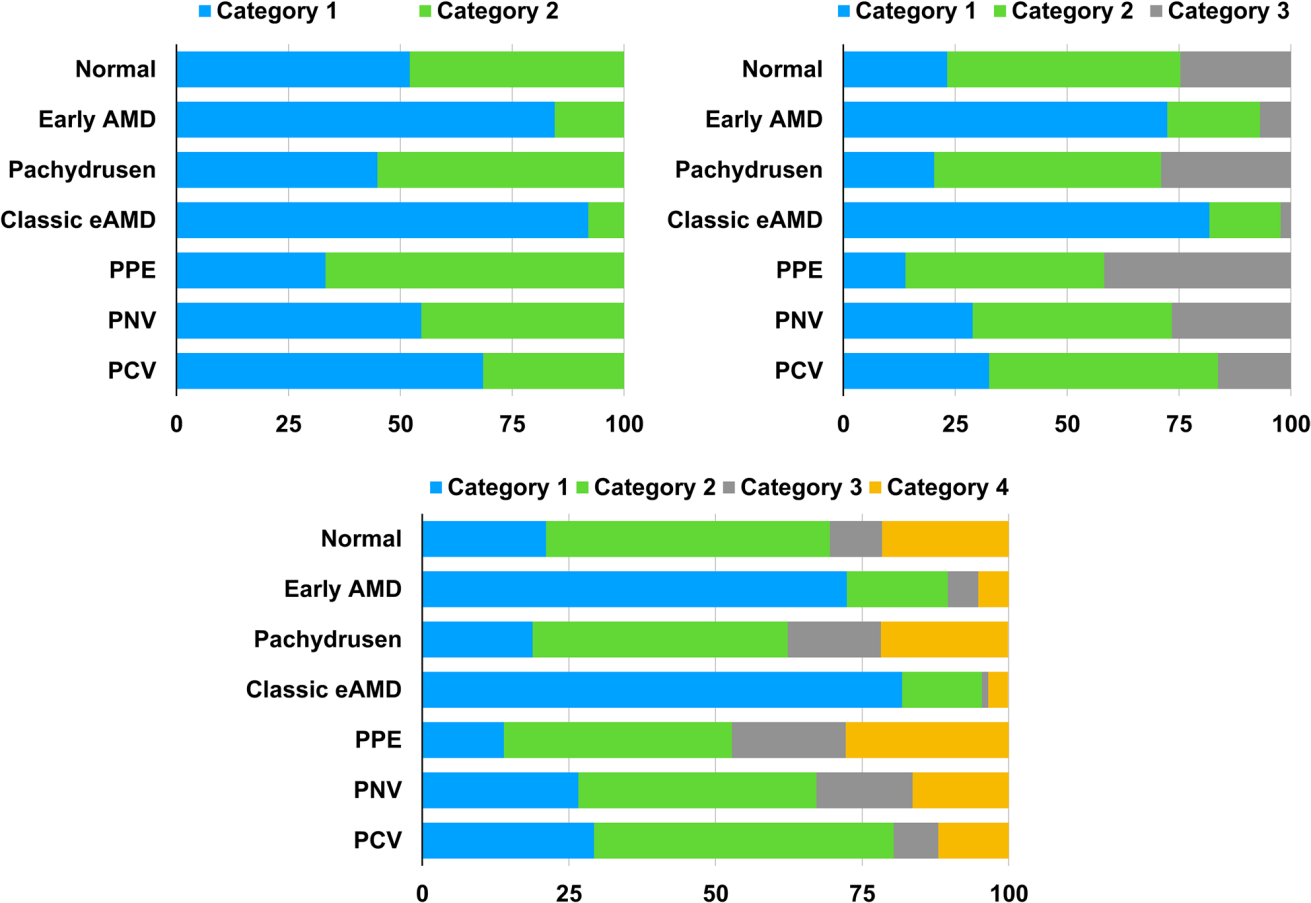
Clustering of eyes with age-related macular degeneration or pachychoroid spectrum diseases among categories based on the choroidal thickness profiles. Early AMD, early AMD with soft drusen or reticular pseudodrusen; eAMD , exudative age-related macular degeneration; PPE, pachychoroid pigment epitheliopathy; PNV, pachychoroid neovasculopathy; PCV, polypoidal choroidal vasculopathy.

**Figure 2:**
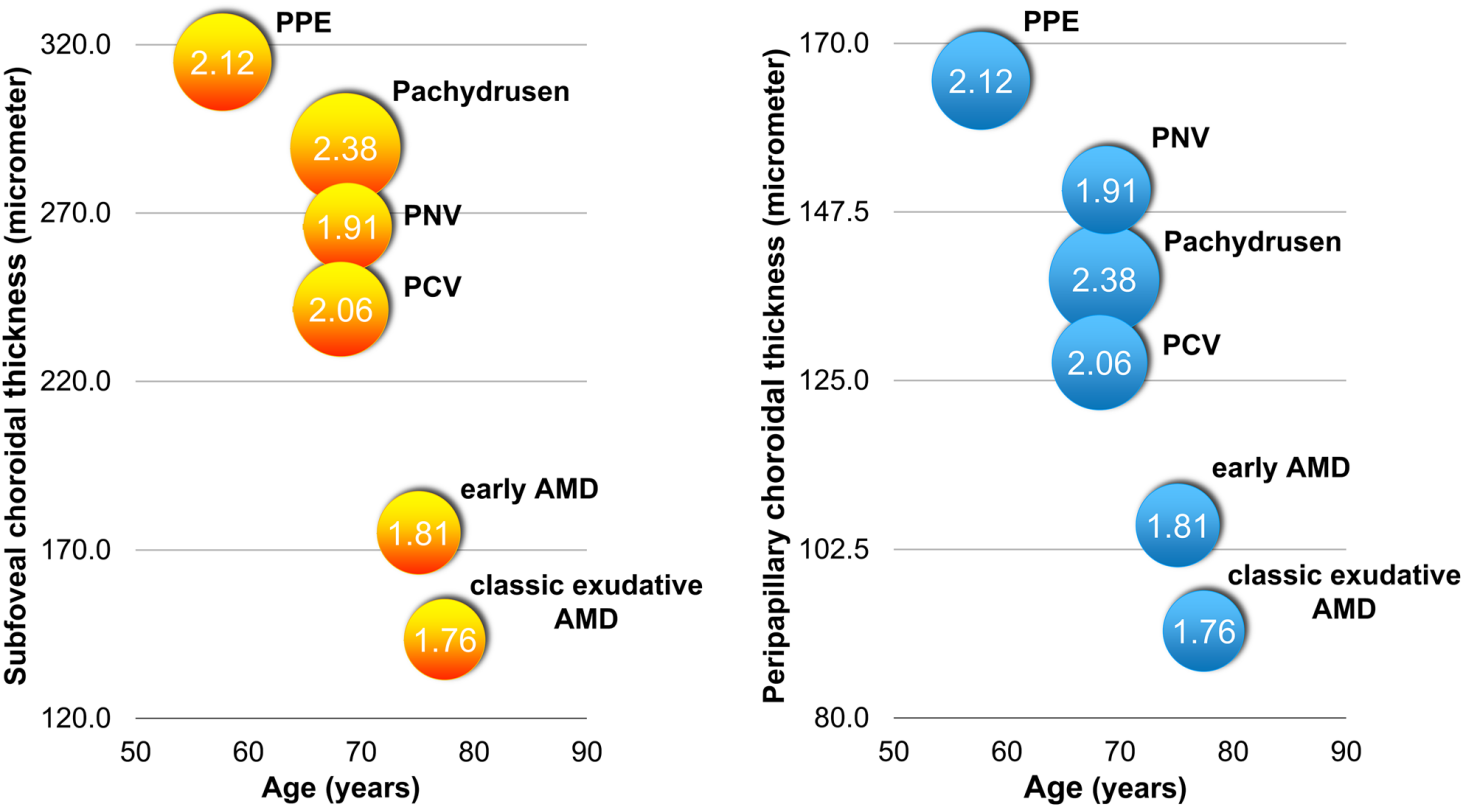
Distribution of age and choroidal profiles including subfoveal choroidal thickness (CT), peripapillary CT and the ratio of subfoveal CT to peripapillary CT according to various groups of age-related macular degeneration and pachychoroid spectrum diseases. The numbers inside the circle represent the ratio of subfoveal CT to peripapillary CT.

Figure 1 shows the distributions of age and choroidal profiles including subfoveal CT, peripapillary CT and the ratio of subfoveal CT to peripapillary CT in various groups of AMD and PSD. And, representative cases of each group are shown in Fig. 2.

In a total 661 eyes, ANCOVA, including age, gender, and groups of AMD or PSDs, showed a correlation between subfoveal and peripapillary CT and age and groups of AMD or PSD (*p* < 0.001, *p* < 0.001; *p* < 0.001, *p* < 0.001, respectively) (Table 4). The ratio of subfoveal CT to peripapillary CT was also significantly affected in the AMD or PSD groups (*p* < 0.001); however, it was independent of age (*p* = 0.168).

**Table 4:** Analysis of covariance for risk factors of change in choroidal thickness profile.

Source of variation	Sum of square	d.f	Mean of squares	F-value	P value
**Subfoveal CT**					
Age	367,122.761	1	367,122.761	43.072	< 0.001
Gender	16,770.227	1	16,770.227	1.968	0.161
Classification of eyes	791,960.199	6	131,993.366	15.486	< 0.001
Classification*Gender	31,097.889	6	5182.982	0.608	0.724
**Peripapillary CT**					
Age	147,047.050	1	147,047.050	44.487	< 0.001
Gender	11,005.552	1	11,005.552	3.330	0.069
Classification of eyes	145,314.615	6	24,219.102	7.327	< 0.001
Classification*Gender	26,704.437	6	4450.739	1.346	0.234
**Ratio of subfoveal CT to peripapillary CT**					
Age	1.370	1	1.370	1.908	0.168
Gender	0.312	1	0.312	0.435	0.510
Classification of eyes	28.679	6	4.780	6.657	< 0.001
Classification*Gender	7.463	6	1.244	1.732	0.111

CT, choroidal thickness.

In addition to age, subfoveal CT, peripapillary CT, and the ratio of subfoveal CT to peripapillary CT was selected for CT profiling.

### Clustering of choroidal thickness profile and comparison of the clustering distribution between groups of age-related macular degeneration and pachychoroid spectrum diseases

Choroidal thickness profiles from 661 eyes were categorized into 2, 3, or 4 clusters (Table 5). When the number of clusters were prespecified to 2, the CT profile was categorized into Cluster 1 with the lowest values of all CT profiles (405 eyes, 61.3%) and Cluster 2 with the greatest values of all CT profiles (256 eyes, 38.7%) (Table 6). When the number of clusters was set to 3, the CT profile was categorized into Cluster 1 with the lowest values of CT profiles (224 eyes, 36.9%),a Cluster 2 with the greatest ratio of subfoveal CT to peripapillary CT (280 eyes, 42.4%), and Cluster 3 with the greatest values among CT profiles, except the ratio of subfoveal CT to peripapillary CT (137 eyes, 20.7%). When the number of clusters was set to 4, the CT profile was classified into Cluster 1 with the lowest values among CT profiles, except the ratio of subfoveal CT to peripapillary CT (233 eyes, 35.2%), Cluster 2 with second greatest ratio of subfoveal CT to peripapillary CT (257 eyes, 38.9%), Cluster 3 with the greatest peripapillary CT and the lowest ratio of subfoveal CT to peripapillary CT (67 eyes, 10.1%), and Cluster 4 with the greatest values among CT profiles, except peripapillary CT (104 eyes, 15.7%).

**Table 5:** Clustering analysis based on choroidal thickness profile and age.

	Number of eyes (%)	Final cluster centers			
		Subfoveal CT	Peripapillary CT	Ratio of subfoveal CT to peripapillary CT	Age
**Number of clusters = 2**					
Cluster 1	405 (61.3%)	179	100	1.96	69
Cluster 2	256 (38.7%)	356	179	2.21	61
*P* value		< 0.001	< 0.001	< 0.001	< 0.001
**Number of clusters = 3**					
Cluster 1	244 (36.9%)	138	86	1.82	72
Cluster 2	280 (42.4%)	267	134	2.22	64
Cluster 3	137 (20.7%)	400	205	2.15	59
*P* value		< 0.001	< 0.001	< 0.001	< 0.001
**Number of clusters = 4**					
Cluster 1	233 (35.2%)	135	86	1.76	72
Cluster 2	257(38.9%)	259	125	2.3	64
Cluster 3	67 (10.1%)	338	247	1.41	61
Cluster 4	104 (15.7%)	413	171	2.55	60
*P* value		< 0.001	< 0.001	< 0.001	< 0.001

CT, choroidal thickness.

**Table 6:** Comparison of the distribution of age-related macular degeneration (AMD) or pachychoroid spectrum disease (PSD) between categories based on the choroidal thickness (CT) profile and age.

Variables	Clustering Number = 2		Clustering Number = 3			Clustering Number = 4			
	Category 1	Category 2	Category 1	Category 2	Category 3	Category 1	Category 2	Category 3	Category 4
Number of eyes (%)	405 (61.3%)	256 (38.7%)	244 (36.9%)	280 (42.4%)	137 (20.7%)	233 (35.2%)	257 (38.9%)	67 (10.1%)	104 (15.7%)
**Final cluster centers**									
Subfoveal CT, μm	179	356	138	267	400	135	259	338	413
Peripapillary CT, μm	100	179	86	134	205	86	125	247	171
Ratio of subfoveal CT to peripapillary CT	1.96	2.21	1.82	2.22	2.15	1.76	2.3	1.41	2.55
Age, years	69	61	72	64	59	72	64	61	60
**Groups of AMD or PSD, n (%)**									
Normal (190 eyes)	99 (52.1%)	91 (47.9%)	44 (23.2%)	99 (52.1%)	47 (24.7%)	40 (21.1%)	92 (48.4%)	17 (8.9%)	41 (21.6%)
Early AMD (58 eyes)	49 (84.5%)	9 (15.5%)	42 (72.4%)	12 (20.7%)	4 (6.9%)	42 (72.4%)	10 (17.2%)	3 (5.2%)	3 (5.2%)
Pachydrusen (69 eyes)	31 ( 44.9%)	38 (55.1%)	14 (20.3%)	35 (50.7%)	20 (29.0%)	13 (18.8%)	30 (43.5%)	11 (15.9%)	15 (21.7%)
Classic exudative AMD (88 eyes)	81 (92.0%)	7 (8.0%)	72 (81.8%)	14 (15.9%)	2 (2.3%)	72 (81.8%)	12 (13.6%)	1 (1.1%)	3 (3.4%)
PPE (36 eyes)	12 (33.3%)	24 (66.7%)	5 (13.9%)	16 (44.4%)	15 (41.7%)	5 (13.9%)	14 (38.9%)	7 (19.4%)	10 (27.8%)
PNV (128 eyes)	70 (54.7%)	58 (45.3%)	37 (28.9%)	57 (44.5%)	34 (26.6%)	34 (26.6%)	52 (40.6%)	21 (16.4%)	21 (16.4%)
PCV (92 eyes)	63 (68.5%)	29 (31.5%)	30 (32.6%)	47 (51.1%)	15 (16.3%)	27 (29.3%)	47 (51.1%)	7 (7.6%)	11 (12.0%)
*P* value	< 0.001		< 0.001			< 0.001			

CT, choroidal thickness; AMD, age-related macular degeneration; PSD, pachychoroid spectrum diseases; early AMD, early AMD with soft drusen or reticular pseudodrusen; PPE, pachychoroid pigment epitheliopathy, PNV, pachychoroid neovasculopathy; PCV, polypoidal choroidal vasculopathy.

**Figure 3:**
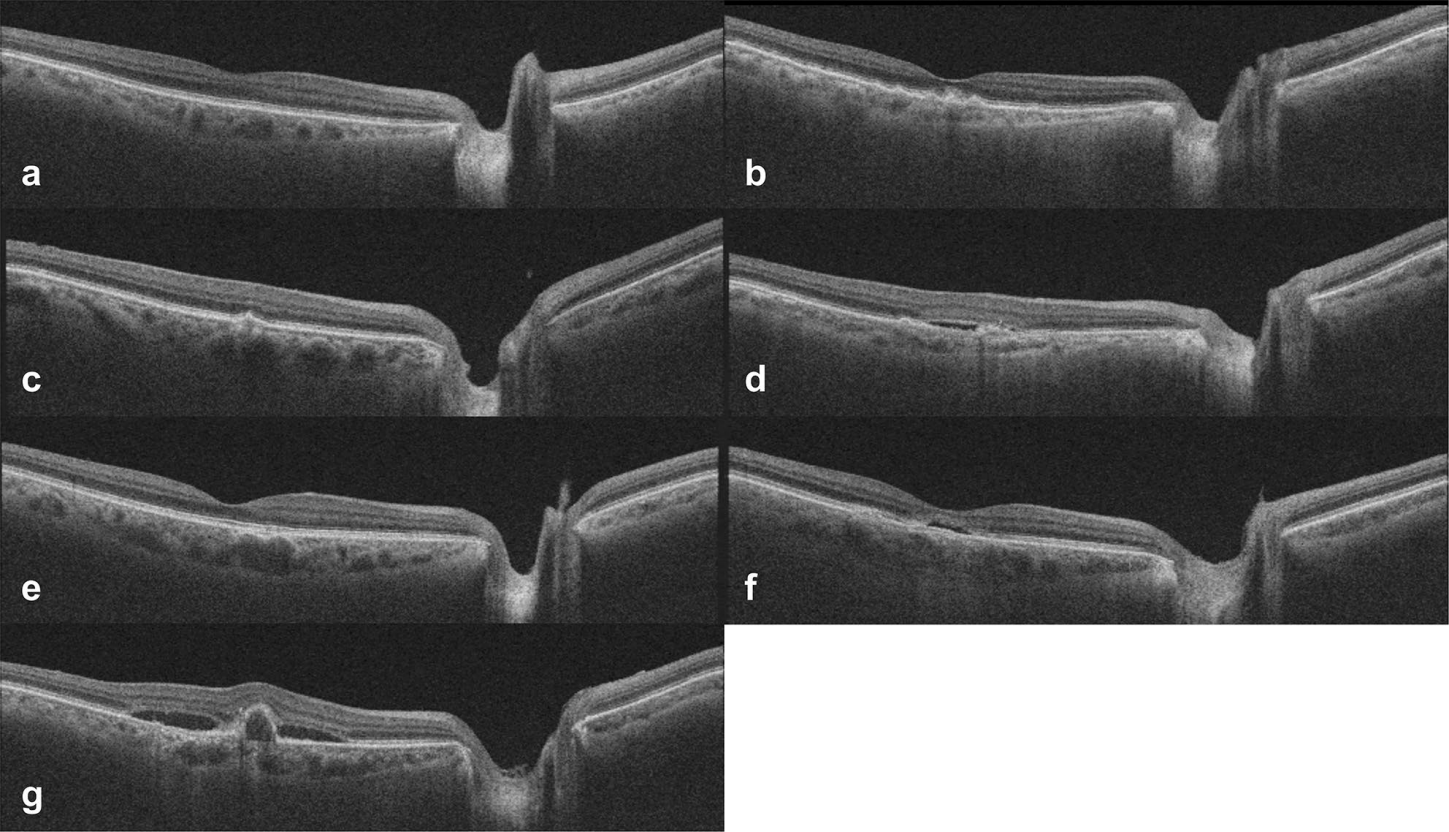
Representative swept-source optical coherence tomography images of eyes with (**a**) normal control, (**b**) early age-related macular degeneration (AMD) with soft drusen or reticular pseudodrusen, (**c**) pachydrusen, (**d**) classic exudative AMD, (**e**) pachychoroid pigment epitheliopathy, (**f**) pachychoroid neovasculopathy, and (**g**) polypoidal choroidal vasculopathy. The subfoveal choroidal thicknesses (CT), peripapillary CTs and the ratios of subfoveal CT to peripapillary CT are (**a**) 287 μm, 117 μm, 2.45, (**b**) 142 μm, 102 μm, 1.39, (**c**) 322 μm, 141 μm, 2.28, (**d**) 143 μm, 78 μm, 1.83, (**e**) 337 μm, 154 μm, 2.19, (**f**) 347 μm, 188 μm, 1.85, and (**g**) 264 μm, 132 μm, 2.00, respectively.

Among the AMD groups, most eyes of the early AMD and classic exudative AMD groups belonged to the same clusters, independent of the number of clusters set to 2, 3, or 4 (all *p* < 0.001) (Fig. 3). However, most eyes in the pachydrusen group were not clustered with eyes of the early AMD or classic exudative AMD groups. They showed distribution patterns of clustering similar to the eyes of the PSD groups (all *p* < 0.001). In the PSD groups, most eyes of the PNV and PCV groups tended to be in the same clusters, independent of the number of clusters set to 2, 3, or 4 (all *p* < 0.001). When the number of clusters were set to 2, the distribution of eyes in the PNV or PCV group was not clearly separated from the cluster of eyes in the early AMD or classic exudative AMD groups. However, when the number of clusters increased to 3 or 4, eyes in the PNV or PCV groups were distinguished in different clusters from eyes in the early AMD or classic exudative AMD groups. Eyes of the PPE group belonged to clusters with the ratio of subfoveal CT to peripapillary CT > 2.0, independent of the number of clusters.

## Discussion

In previous studies, AMD or PSD was classified according to the characteristics of lesions occurring in the retina and choroid, and CT was compared between these classifications^7,30–34^. However, few studies have investigated the distribution of lesions of the retina after categorizing the CT profile. In this study, we classified eyes based on the choroidal profiles regardless of fundus findings. Additionally, we tried to find out the clustering patterns of each disease group. The eyes with pachydrusen had a greater tendency to be clustered with eyes of the PSD groups than with other AMD groups. Among the diseases in which MNV occurs, it was also confirmed that PNV and PCV tended to be in the same cluster, whereas classic exudative AMD belongs to a different cluster than PNV, PCV and other clusters with typical early AMD characterized by soft drusen. The results of the current study could not confirm whether there is a difference in the mechanism of MNV in these neovascular diseases. However, the finding that these diseases are classified differently in clusters based on the CT profile supports the suggestion that the choroid will contribute differently to the development of these diseases.

Drusen is a hallmark of AMD, which occurs in the Bruch’s membrane and RPE^2,35,36^. Drusen in AMD has been classified into hard drusen and soft drusen^36,37^. Recently, it has been suggested that pachydrusen has different characteristics from typical soft drusen^35,38,39^. Several previous studies showed that the progression of AMD was different between eyes with soft drusen and pachydrusen^30,39^. Although aging can contribute to the development of both types of drusen^30,35,40^, it is unclear how it influences eyes with thin and thick choroid. In the current study, we isolated pachydrusen and compared characteristics between patients with soft drusen and pachydrusen. The average age of patients with soft drusen was 75.1 years, which was greater than those with pachydrusen. It could mean that differences in age affect the development of pachydrusen and soft drusen. In our study, the group with pachydrusen had a thicker CT profile compared to AMD groups, which is consistent with previous studies^35,38,40^. Recent findings that pachyvessel is a general phenomenon of PSDs and pachydrusen, and that focal ischemia caused by pachyvessels affects the development of these lesions, suggests that pachydrusen may have the common pathogenesis as PSDs^5,14,40,41^. Indeed, the finding of the current study that eyes with pachydrusen had a greater tendency to be clustered with eyes of PSD groups than with the AMD groups with soft drusen or exudation could support this suggestion. A previous study^39^ reported that the incidence of MNV was significantly lower in fellow eyes with pachydrusen than in fellow eyes with soft drusen or reticular pseudodrusen in Asian patients with unilateral MNV. Additionally, the result of the current study suggests that pachydrusen may be classified as a class of PSD rather than classic AMD. Given the suggestion that age may affect some PSDs, such as PPE, PNV, and PCV, pachydrusen can be classified into the same group as the age-related PSDs or age-related choroidal vasculopathy compared to classic AMD^42^.

To determine the CT profile which could represent the choroidal status in eyes with AMD or PSDs, we compared various CT parameters among eyes with and without AMD or PSDs. We found that subfoveal CT, peripapillary CT, and the ratio of subfoveal CT to peripapillary CT were significantly different among eyes with AMD and PSDs. We also found that age is a significant factor that contributes to this difference, except the ratio of subfoveal CT to peripapillary CT. Additionally, we defined CT profile, including subfoveal CT, peripapillary CT and the ratio of subfoveal CT to peripapillary CT. There have been many studies of CT in AMD and PSD^12,30–34^. In previous studies, it was reported that there was no difference between patients with AMD and normal subjects, except in the case of much progression, such as geographic atrophy^32^. However, others have reported that the subfoveal CT varies according to the classification of macular drusen^29,35,43^. Meanwhile, in patients with PSD, an increase in subfoveal CT was reported compared to normal subjects^31–33,44^. PSD has been classified into several types^5,6^, and the change in CT based on these classifications have also been reported^14,31,33,39^. There were many studies comparing subfoveal CT between these diseases^14,30,31,33,34,38^, but few studies^32^ compared various stages of AMD and PSDs in one model. In this study, we compared differences in CT with multiple groups of AMD and PSDs. In this study, we found that AMD eyes with soft drusen or classic exudation have a CT profile different from that of the normal group, as well as the PSD group. This result is inconsistent with previous studies^32^. In our previous study^32^, we were unable to find a significant difference between the subfoveal CT in patients with normal fundus and wet AMD. This difference may be due to the fact that in this study we were better able to measure thicker choroids using SS-OCT^45,46^. It may also be due to the fact that in this study, we classified PNV separately, since some eyes that were previously classified as classic exudative AMD were classified as PNV^5,9,47^. Although CT was not used in the PNV diagnostic criteria in this study, it ay be due to the fact that PNV has a thicker CT than classic exudative AMD. In the PSD group, all subfoveal CTs were not significantly different from normal controls; this result is different from those of previous studies^31,32^. In previous studies, some authors reported that patients with PCV had increased subfoveal CT than normal controls^32,48^. However, other authors reported that the subfoveal CT of patients with PCV showed wide range of distribution and significant overlap with classic exudative AMD and normal subjects^49,50^. In addition, several studies showed a bimodal distribution^51–53^. In our study, age-adjusted values were used and there was no difference in subfoveal CT between the PCV group and normal controls.

There have been few studies comparing CT differences between groups using different profiles, such as peripapillary CT and the ratio of subfoveal CT to peripapillary CT, other than subfoveal CT^23,54^. In this study, we compared the peripapillary CT between groups. In previous studies^29^, in addition to subfoveal CT, peripapillary CT was proposed as indicator of choroidal properties. The distribution of peripapillary CT between groups showed a different pattern from subfoveal CT. The peripapillary CT of the AMD groups was not different from normal controls, whereas the subfoveal CT of the early AMD and classic exudative AMD groups were reduced compared to the normal controls. This means that changes in CT involved in AMD are mainly related to changes in the choroid of the macula. Although AMD occurs in the macula^55^, there has been controversy over the sequential relationship between AMD and choroidal changes^56–59^. In this study, the difference in thickness of the choroid outside the macula was not substantial. The fact that AMD is not significantly altered outside the macula supports the claim that AMD and macular choroidal changes are correlated. Meanwhile, there were some differences in the distribution of subfoveal and peripapillary CT in the PSD group. All subfoveal CTs were not different from normal controls; however, peripapillary CT of PNV was thicker than normal controls. This is the result of supporting the suggestion that peripapillary CT may be useful for exhibiting choroidal properties in addition to subfoveal CT^22,23^. Indeed, subfoveal and peripapillary CT were correlated, and they all were correlated with age. However, the ratio of subfoveal CT to peripapillary CT was not related to age or gender. This means that the ratio is generally maintained at a constant level. It has not been well known about the ratio of subfoveal CT to peripapillary CT. In this study, we found that the ratio was calculated as 2.30 in the normal population based on age 66, and this result would be another useful surrogate marker for choroidal status. The distribution of the ratio of subfoveal CT to peripapillary CT among groups showed slightly different patterns from those of subfoveal CT or peripapillary CT. As with subfoveal CT, the ratio of subfoveal CT to peripapillary CT in the early AMD and classic exudative AMD groups was less than those in normal controls. This is a result confirming that the CT reduction of the macula in these two groups is more severe than that outside of the macula. On the other hand, the ratio was greater in PNV than in normal controls, indicating that PNV has different characteristics from other diseases, along with increased peripapillary CT in PNV^16^. The use of these factors in addition to subfoveal CT, including differences in the proportions of subfoveal and peripapillary CT, may help elucidate the nature of the disease.

There are several limitations in this study. This study is a large-scale study involving hundreds of people. However, the number in each group is not the same. Additionally, age was not the same between groups. Although we tried to minimize the effects of these differences using statistical analysis methods, there is still a limitation that the number of each group is not large. In this study, we used fundus photography (FP) and OCT findings for the diagnosis of drusen and used the evaluation and consensus of independent researchers for the classification of drusen. However, the limitation persisted regarding the lack of an absolute standard for classifying soft drusen and pachydrusen. Because of the cross-sectional nature of this study, it does not provide longitudinal information about whether each disease group will retain or change its characteristics of choroid. For PSDs, we only included eyes with PPE, PNV, or PCV. But we did not include other types of PSD such as peripapillary pachychoroid syndrome, focal choroidal excavation, or CSC. Therefore, the results of this study may not be applicable to all PSDs. We included only one symptomatic eye for each patient. It was not confirmed whether the results of this study could be applied to contralateral eyes of patients. Further research is required.

In conclusion, we showed that CT profiling using subfoveal CT, peripapillary CT, and the ratio of subfoveal CT to peripapillary CT was useful to show a variation in CT at the macula and outside of the macula in eyes with various degrees of AMD and PSDs. Classification of the CT profile showed specific clustering patterns among various AMD or PSD groups. However, eyes with pachydrusen were not clustered with eyes in other AMD groups; they were clustered with those in the PSD groups. Therefore, CT profiling may help determine the effect of choroid on the development and progression of AMD and PSDs.

## Methods

The Institutional Review Board of Korea University approved this study (IRB number: 2020AN0280), and all research and data collection were conducted in accordance with the tenets of the Declaration of Helsinki.

In this retrospective cross-sectional comparative study, patients with AMD or PSDs in the SS-OCT database were reviewed between March 2016 and May 2020 at the Korea University Medical Center. From the database, we included eyes with AMD or PSDs. For age-related PSDs, we only included eyes with PPE, PNV, or PCV. We also included a group of normal controls who had eyes with bilateral normal fundus or eyes with epiretinal membrane in their fellow eyes. In cases with MNV in one eye and drusen in the contralateral eye, we selected eyes with MNV. Only one eye per patient was selected for this study in other groups of AMD or PSDs. Other exclusion criteria were as follows: eyes with disciform scar or center involving geographic atrophy, eyes with both soft drusen and pachydrusen, patients who underwent photodynamic therapy, laser photocoagulation or an intraocular surgery, except cataract surgery, and prior history of intravitreal injection of anti-vascular endothelial growth factor within 6 months.

All normal subjects underwent FP, and SS-OCT (DRI OCT Triton, software version 10.10; Topcon Corp., Tokyo, Japan). And, all patients with soft drusen, reticular pseudodrusen, pachydrusen and PPE underwent FP, fundus autofluorescence (FAF), near infrared reflectance (NIR) images and SS-OCT and SS-OCT angiography. All patients with MNV also underwent fluorescein angiography (FA) and indocyanine green angiography (ICG) (Spectralis HRA; Heidelberg Engineering, Heidelberg, Germany). Classification of neovascular lesions and drusen was performed independently by two retina specialists (Y.K. and B.L.) who evaluated the FP, FAF, NIR, OCT, OCTA, FA, and ICG images.

### Classification of age-related macular degeneration and pachychoroid spectrum diseases

The extracellular deposits (drusen) were determined using FP and SS-OCT, based on the criteria used in previous studies^35,38^, and they were classified as reticular pseudodrusen, soft drusen, and pachydrusen. Subsequently, the eyes with various types of drusen were grouped into early AMD group with soft drusen or reticular pseudodrusen^60^ and group with pachydrusen^39,61^. Eyes with MNV were classified as a classic exudative AMD group.

We classified eyes with PSDs into PPE, PNV, and PCV groups based on previous studies. PPE was determined when eyes had PPE with characteristic RPE alterations, except pachydrusen, and either a focally or diffusely thickened choroid seen with SS-OCT^44,62^. We defined PNV by modifying the definitions from previous studies^5,34^ and diagnosed these cases after reviewing the medical records of patients with neovascular AMD using the following criteria: (1) definite neovascularization on OCT and ancillary images, including OCTA and FA/ICG images; (2) no soft drusen or reticular pseudodrusen in both eyes (no AMD, AREDS category 1); (3) with or without pachydrusen in either eye; (4) PPE or CSC characteristics: choroidal vascular hyperpermeability on ICG, the presence of dilated outer choroidal vessels (pachyvessels) with attenuation, and thinning of the choriocapillaris and Sattler’s vessels, or a history of CSC. PCV was diagnosed by ICG showing single or multiple polypoidal aneurysmal bulges with or without a branching vascular network. A third supervising grader (J.O.) evaluated the lesion type in the presence of significant discrepancies.

### Measurement of choroidal thickness

**Figure 4:**
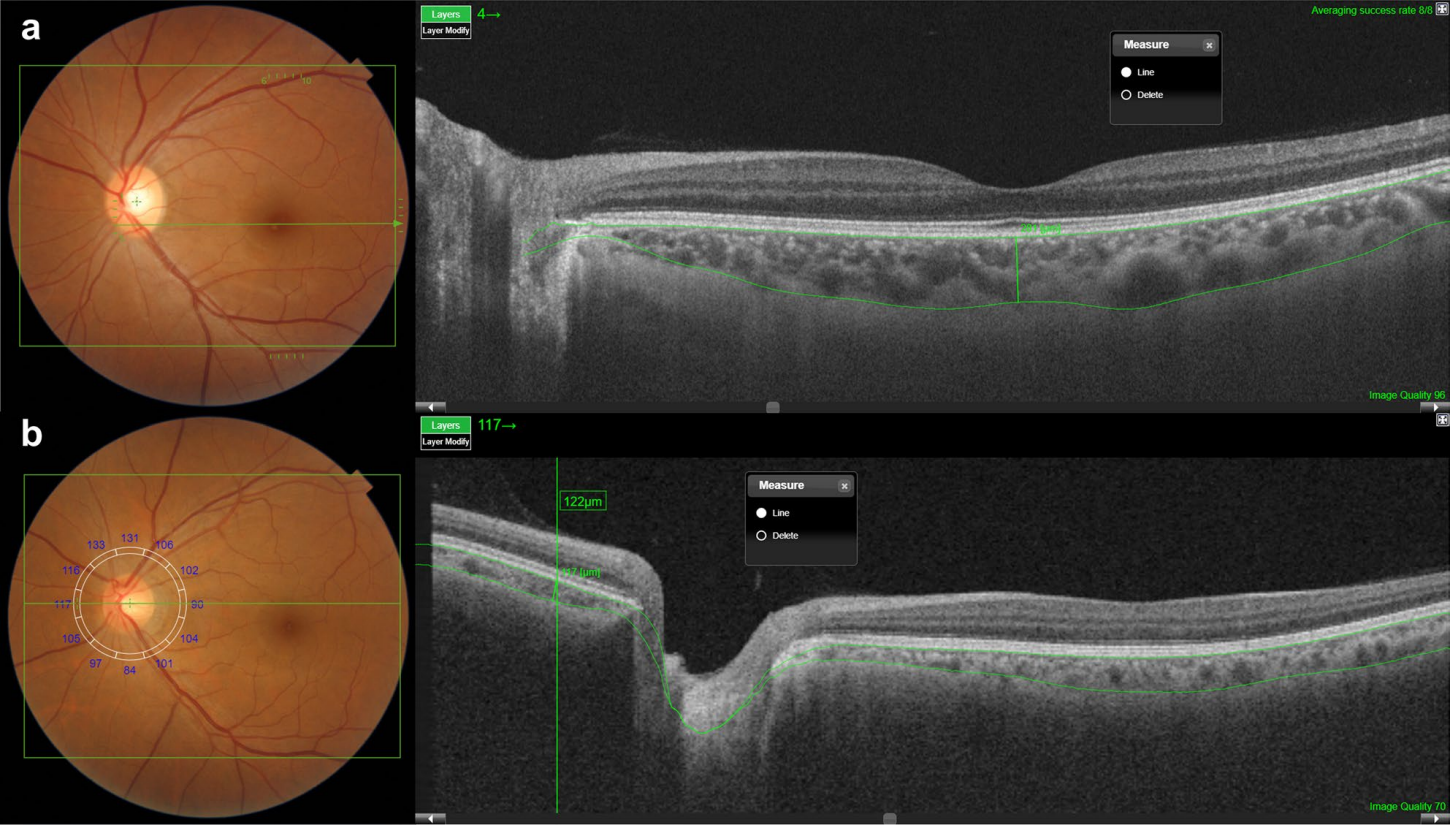
The methods for subfoveal and peripapillary choroidal thickness (CT) measurements. (**a**) Subfoveal CT was measured from a 9-mm line scan, which was centered on the fovea and averaged 96 B-scans to improve the signal-to-noise ratio. (**b**) Peripapillary CT was measured from a 12-mm by a 9-mm volume scan image, which was centered between the fovea and the optic disc.

The CT was defined as the perpendicular distance from the inner surface of the RPE to the choroidoscleral interface. Using a built-in image viewer program of SS-OCT, CT was measured manually by caliper tool. By previously reported method^63^, the subfoveal CT and peripapillary CT were measured to represent the CT in the macular area and CT on the outside of the macular area, respectively. We measured subfoveal CT from a 9-mm line scan, which was centered on the fovea and averaged 96 B-scans to improve the signal-to-noise ratio. Additionally, we measured peripapillary CT from a 12-mm by a 9-mm volume scan image, which was centered between the fovea and the optic disc (Fig. 4). In the volume scan, we measured the peripapillary CT manually at the nasal point of the 360° 3.4-mm-diameter circle that was centered on the optic disc to measure the retinal nerve fiber layer. Two independent observers (Y.K. and B.L.) performed all measurements without patient information. The interobserver intraclass correlation coefficients (ICC) for the subfoveal CT and peripapillary CT were 0.982 (95% confidence interval, 0.979–0.984) and 0.975 (95% confidence interval, 0.970–0.978), respectively. Both ICCs were greater than 0.90 and showed excellent repeatability. The mean value of the two measurements was used for the final analysis.

### Determination of choroidal thickness profile

To determine the parameters comprising the CT profile, we compared various parameters, including age, gender, subfoveal CT, peripapillary CT, and the ratio of subfoveal CT to peripapillary CT between AMD and PSD groups. Additionally, we determined the factors that comprise the CT profile. We compared the characteristics of the groups by using the independent t-test for continuous variables, and the chi-square test for categorical variables. We analyzed linear correlations with Pearson’s correlation coefficient (*r*) for normally distributed continuous variables. Analyses of covariance (ANCOVA) with adjustments for age and gender were performed to assess differences in the measurements between groups. In the analysis of covariance tests, we estimated marginal means of CT as calculated and compared between groups. We considered results to be statistically significant at *p* values < 0.05. All statistical analyses were performed using SPSS software version 20.0 (IBM corp., Armonk, NY, USA).

### Clustering of choroidal thickness profiles and analysis of the distribution of eyes with AMD and PSDs

After determining the factors comprising the CT profiles, we classified eyes into several clusters by using clustering analysis with the k-means method in SPSS software. The number of clusters were prespecified into sets of 2, 3, or 4. Additionally, we compared the distribution of eyes between various clusters with different AMD or PSD based on the CT profile.

### Ethics approval

This study was performed in line with the principles of the Declaration of Helsinki and approved by the Institutional Review Board of Korea University Hospital, Seoul, Korea (IRB number: 2020AN0280), and adhered to the tenets of the Declaration of Helsinki.

### Consent to participate

This retrospective study involves no more than minimal risk to subjects and the IRB of Korea University Hospital approved our request to waive of informed consent.

## Supplementary Information

Supplementary Information

## Data Availability

All data generated or analysed during this study are included in this published article (and its Supplementary Information files). The datasets generated during and/or analysed during the current study are available from the corresponding author on reasonable request.

## References

[1] Ardeljan D, Chan CC (2013). Aging is not a disease: distinguishing age-related macular degeneration from aging. Prog. Retin. Eye Res..

[2] Coleman HR, Chan CC, Ferris FL, Chew EY (2008). Age-related macular degeneration. Lancet.

[3] Yanagi Y (2020). Pachychoroid disease: a new perspective on exudative maculopathy. Jpn. J. Ophthalmol..

[4] Cheung CMG (2019). Pachychoroid disease. Eye..

[5] Pang CE, Freund KB (2015). Pachychoroid neovasculopathy. Retina..

[6] Siedlecki J, Schworm B, Priglinger SG (2019). The pachychoroid disease spectrum-and the need for a uniform classification system. Ophthalmol. Retina..

[7] Wong CW (2016). Age-related macular degeneration and polypoidal choroidal vasculopathy in Asians. Prog. Retin. Eye Res..

[8] Balaratnasingam C (2016). Polypoidal choroidal vasculopathy: a distinct disease or manifestation of many?. Retina..

[9] Borrelli E (2020). Rate of misdiagnosis and clinical usefulness of the correct diagnosis in exudative neovascular maculopathy secondary to AMD versus pachychoroid disease. Sci Rep..

[10] Hayreh SS (1990). In vivo choroidal circulation and its watershed zones. Eye..

[11] Nickla DL, Wallman J (2010). The multifunctional choroid. Prog. Retin. Eye Res..

[12] Ting DS (2016). Choroidal thickness changes in age-related macular degeneration and polypoidal choroidal vasculopathy: A 12-month prospective study. Am. J. Ophthalmol..

[13] Adhi M, Duker JS (2013). Optical coherence tomography–current and future applications. Curr. Opin. Ophthalmol..

[14] Dansingani KK, Balaratnasingam C, Naysan J, Freund KB (2016). En Face imaging of pachychoroid spectrum disorders with swept-source optical coherence tomography. Retina..

[15] Spaide RF (2009). Enhanced depth imaging optical coherence tomography of retinal pigment epithelial detachment in age-related macular degeneration. Am. J. Ophthalmol..

[16] Gupta MP (2016). Pachychoroid neovasculopathy in extramacular choroidal neovascularization. Clin. Ophthalmol..

[17] Phasukkijwatana N (2018). Peripapillary pachychoroid syndrome. Retina..

[18] Seddon JM, Reynolds R, Rosner B (2009). Peripheral retinal drusen and reticular pigment: association with CFHY402H and CFHrs1410996 genotypes in family and twin studies. Invest. Ophthalmol. Vis. Sci..

[19] Yannuzzi LA (1998). Idiopathic polypoidal choroidal vasculopathy: a peripheral lesion. Arch. Ophthalmol..

[20] Chakraborty R, Read SA, Collins MJ (2011). Diurnal variations in axial length, choroidal thickness, intraocular pressure, and ocular biometrics. Invest. Ophthalmol. Vis. Sci..

[21] Usui S (2012). Circadian changes in subfoveal choroidal thickness and the relationship with circulatory factors in healthy subjects. Invest. Ophthalmol. Vis. Sci..

[22] Oh J (2013). Simplified method to measure the peripapillary choroidal thickness using three-dimensional optical coherence tomography. Korean J. Ophthalmol..

[23] Yun C (2016). Peripapillary choroidal thickness in patients with early age-related macular degeneration and reticular pseudodrusen. Graefes Arch. Clin. Exp. Ophthalmol..

[24] Chang IB, Lee JH, Kim JS (2017). Changes in choroidal thickness in and outside the macula after hemodialysis in patients with end-stage renal disease. Retina..

[25] Ho J (2011). Analysis of normal peripapillary choroidal thickness via spectral domain optical coherence tomography. Ophthalmology.

[26] Ozcimen M (2016). Peripapillary choroidal thickness in patients with chronic obstructive pulmonary disease. Cutan. Ocul. Toxicol..

[27] Lee KH, Kim SH, Lee JM, Kang EC, Koh HJ (2017). Peripapillary choroidal thickness change of polypoidal choroidal vasculopathy after anti-vascular endothelial growth factor. Korean J. Ophthalmol..

[28] Liu, S. *et al.* Correlation between renal function and peripapillary choroidal thickness in treatment-naïve diabetic eyes using swept-source optical coherence tomography. *Curr. Eye Res.* 1–8 (2020). 10.1080/02713683.2020.1753213 32255371

[29] Yun C (2015). Peripapillary choroidal thickness in central serous chorioretinopathy: is choroid outside the macula also thick?. Retina..

[30] Cheung CMG, Gan A, Yanagi Y, Wong TY, Spaide R (2018). association between choroidal thickness and drusen subtypes in age-related macular degeneration. Ophthalmol. Retina..

[31] Chung SE, Kang SW, Lee JH, Kim YT (2011). Choroidal thickness in polypoidal choroidal vasculopathy and exudative age-related macular degeneration. Ophthalmology.

[32] Kim SW, Oh J, Kwon SS, Yoo J, Huh K (2011). Comparison of choroidal thickness among patients with healthy eyes, early age-related maculopathy, neovascular age-related macular degeneration, central serous chorioretinopathy, and polypoidal choroidal vasculopathy. Retina..

[33] Koizumi H, Yamagishi T, Yamazaki T, Kawasaki R, Kinoshita S (2011). Subfoveal choroidal thickness in typical age-related macular degeneration and polypoidal choroidal vasculopathy. Graefes Arch. Clin. Exp. Ophthalmol..

[34] Miyake M (2015). Pachychoroid neovasculopathy and age-related macular degeneration. Sci. Rep..

[35] Spaide RF (2018). Disease expression in nonexudative age-related macular degeneration varies with choroidal thickness. Retina..

[36] Jager RD, Mieler WF, Miller JW (2008). Age-related macular degeneration. N. Engl. J. Med..

[37] Abdelsalam A, Del Priore L, Zarbin MA (1999). Drusen in age-related macular degeneration: pathogenesis, natural course, and laser photocoagulation-induced regression. Surv. Ophthalmol..

[38] Lee J, Byeon SH (2019). Prevalence and clinical characteristics of pachydrusen in polypoidal choroidal vasculopathy: multimodal image study. Retina..

[39] Lee J (2019). Neovascularization in fellow eye of unilateral neovascular age-related macular degeneration according to different drusen types. Am. J. Ophthalmol..

[40] Matsumoto H (2019). Clinical characteristics of pachydrusen in central serous chorioretinopathy. Graefes Arch. Clin. Exp. Ophthalmol..

[41] Baek J, Kook L, Lee WK (2019). Choriocapillaris flow impairments in association with pachyvessel in early stages of pachychoroid. Sci. Rep..

[42] Lee M, Lee H, Kim HC, Chung H (2018). Changes in stromal and luminal areas of the choroid in pachychoroid diseases: insights into the pathophysiology of pachychoroid diseases. Invest. Ophthalmol. Vis. Sci..

[43] Lee J (2020). Drusen subtypes and choroidal characteristics in asian eyes with typical neovascular age-related macular degeneration. Retina..

[44] Sakurada Y (2020). Relationship between choroidal vascular hyperpermeability, choriocapillaris flow density, and choroidal thickness in eyes with pachychoroid pigment epitheliopathy. Retina..

[45] Copete S, Flores-Moreno I, Montero JA, Duker JS, Ruiz-Moreno JM (2014). Direct comparison of spectral-domain and swept-source OCT in the measurement of choroidal thickness in normal eyes. Br. J. Ophthalmol..

[46] Lee MW (2020). Comparison of choroidal thickness measurements using swept source and spectral domain optical coherence tomography in pachychoroid diseases. PLoS ONE.

[47] Fung AT, Yannuzzi LA, Freund KB (2012). Type 1 (sub-retinal pigment epithelial) neovascularization in central serous chorioretinopathy masquerading as neovascular age-related macular degeneration. Retina..

[48] Lee K, Park JH, Park YG, Park YH (2020). Analysis of choroidal thickness and vascularity in patients with unilateral polypoidal choroidal vasculopathy. Graefes Arch. Clin. Exp. Ophthalmol..

[49] Gupta P (2017). Detailed characterization of choroidal morphologic and vascular features in age-related macular degeneration and polypoidal choroidal vasculopathy. Retina..

[50] Kong M, Kim SM, Ham DI (2017). Comparison of clinical features and 3-month treatment response among three different choroidal thickness groups in polypoidal choroidal vasculopathy. PLoS ONE.

[51] Lee WK, Baek J, Dansingani KK, Lee JH, Freund KB (2016). Choroidal morphology in eyes with polypoidal choroidal vasculopathy and normal or subnormal subfoveal choroidal thickness. Retina..

[52] Jordan-Yu JM (2020). Polypoidal choroidal vasculopathy features vary according to sub-foveal choroidal thickness. Retina..

[53] Chang YC, Cheng CK (2020). Difference between pachychoroid and nonpachychoroid polypoidal choroidal vasculopathy and their response to anti-vascular endothelial growth factor therapy. Retina..

[54] Nam KT (2020). Features of the macular and peripapillary choroid and choriocapillaris in eyes with nonexudative age-related macular degeneration. Retina..

[55] Ferris FL (2013). Clinical classification of age-related macular degeneration. Ophthalmology.

[56] Xu W (2010). Association of risk factors for choroidal neovascularization in age-related macular degeneration with decreased foveolar choroidal circulation. Am. J. Ophthalmol..

[57] Chirco KR, Sohn EH, Stone EM, Tucker BA, Mullins RF (2017). Structural and molecular changes in the aging choroid: implications for age-related macular degeneration. Eye..

[58] Zarbin MA (2004). Current concepts in the pathogenesis of age-related macular degeneration. Arch Ophthalmol.

[59] Boltz A (2010). Choroidal blood flow and progression of age-related macular degeneration in the fellow eye in patients with unilateral choroidal neovascularization. Invest. Ophthalmol. Vis. Sci..

[60] Zweifel SA, Imamura Y, Spaide TC, Fujiwara T, Spaide RF (2010). Prevalence and significance of subretinal drusenoid deposits (reticular pseudodrusen) in age-related macular degeneration. Ophthalmology.

[61] Fukuda Y (2019). Clinical and genetic characteristics of pachydrusen in patients with exudative age-related macular degeneration. Sci. Rep..

[62] Warrow DJ, Hoang QV, Freund KB (2013). Pachychoroid pigment epitheliopathy. Retina..

[63] Kim YH, Lee B, Kang E, Oh J (2021). Choroidal thickness profile and clinical outcomes in eyes with polypoidal choroidal vasculopathy. Graefes Arch. Clin. Exp. Ophthalmol..

