# Supplementary material for: Clustering of eyes with age-related macular degeneration or pachychoroid spectrum diseases based on choroidal thickness profile
Source: Sci Rep. 2021 Mar 2;11:4999. doi: 10.1038/s41598-021-84650-7 (PMC7925534; doi:10.1038/s41598-021-84650-7)
Supplement: Supplementary file 1 — Supplementary Information [file 41598_2021_84650_MOESM1_ESM.pdf]

Clustering of eyes with age-related macular degeneration or pachychoroid spectrum diseases based on choroidal thickness profile

Young Ho Kim, Boram Lee, Edward Kang, Jaeryung Oh

Department of Ophthalmology, Korea University Medicine, Seoul, Korea

Supplementary Table S1. *Post hoc* multiple pairwise comparisons of subfoveal choroidal thickness, peripapillary choroidal thickness and ratio of subfoveal to peripapillary choroidal thickness based on estimated marginal means.

Groups		Subfoveal CT				Peripapillary CT				Ratio of subfoveal to peripapillary CT			
(I) Group	(J) Group	Mean Difference (I-J)	95% CI for Difference Lower Bound	95% CI for Difference Upper Bound	*P* value	Mean Difference (I-J)	95% CI for Difference Lower Bound	95% CI for Difference Upper Bound	Significance	Mean Difference (I-J)	95% CI for Difference Lower Bound	95% CI for Difference Upper Bound	*P* value
Normal control	eAMDd	63.6	12.1	115.2	0.004	-2.2	-34.3	29.9	1.000	0.6	0.2	1.1	0.001
	eAMDpd	-39.0	-81.0	3.0	0.100	-21.0	-47.2	5.1	0.301	-0.1	-0.5	0.3	1.000
	ceAMD	84.5	40.9	128.1	0.000	13.5	-13.6	40.6	1.000	0.6	0.2	1.0	0.001
	PPE	-38.9	-92.1	14.2	0.540	-26.4	-59.5	6.7	0.320	0.1	-0.4	0.6	1.000
	PNV	-11.4	-48.0	25.2	1.000	-33.4	-56.2	-10.6	0.000	0.4	0.1	0.8	0.002
	PCV	13.6	-26.9	54.0	1.000	-7.5	-32.7	17.7	1.000	0.2	-0.1	0.6	1.000
eAMDd	eAMDpd	-102.6	-157.4	-47.9	0.000	-18.9	-53.0	15.2	1.000	-0.7	-1.2	-0.2	0.000
	ceAMD	20.9	-31.5	73.3	1.000	15.7	-17.0	48.3	1.000	-0.1	-0.6	0.4	1.000
	PPE	-102.5	-169.8	-35.3	0.000	-24.2	-66.1	17.7	1.000	-0.6	-1.2	0.1	0.114
	PNV	-75.0	-125.4	-24.5	0.000	-31.2	-62.7	0.2	0.053	-0.2	-0.7	0.3	1.000
	PCV	-50.1	-103.8	3.7	0.098	-5.3	-38.8	28.2	1.000	-0.4	-0.9	0.1	0.221
eAMDpd	ceAMD	123.5	76.6	170.4	0.000	34.5	5.3	63.8	0.007	0.6	0.2	1.1	0.000
	PPE	0.1	-60.5	60.6	1.000	-5.3	-43.0	32.4	1.000	0.1	-0.4	0.7	1.000
	PNV	27.6	-15.6	70.8	1.000	-12.4	-39.3	14.5	1.000	0.5	0.1	0.9	0.003
	PCV	52.6	5.7	99.4	0.014	13.6	-15.6	42.7	1.000	0.3	-0.1	0.7	0.739
ceAMD	PPE	-123.4	-184.7	-62.2	0.000	-39.9	-78.0	-1.7	0.032	-0.5	-1.0	0.1	0.214
	PNV	-95.9	-137.7	-54.1	0.000	-46.9	-72.9	-20.9	0.000	-0.1	-0.5	0.3	1.000
	PCV	-71.0	-116.7	-25.2	0.000	-21.0	-49.5	7.5	0.527	-0.3	-0.7	0.1	0.382
PPE	PNV	27.6	-29.3	84.4	1.000	-7.0	-42.5	28.4	1.000	0.4	-0.2	0.9	0.834
	PCV	52.5	-7.0	112.0	0.153	18.9	-18.1	55.9	1.000	0.1	-0.4	0.7	1.000
PVN	PCV	24.9	-16.9	66.8	1.000	25.9	-0.1	52.0	0.053	-0.2	-0.6	0.2	1.000

CT, choroidal thickness; CI, confidence interval; eAMDd, early AMD with soft drusen or reticular pseudodrusen; eAMDpd, early AMD with pachydrusen; ceAMD, classic exudative age-related macular degeneration; PPE, pachychoroid pigment epitheliopathy, PNV, pachychoroid neovasculopathy; PCV, polypoidal choroidal vasculopathy

*Bonferroni adjustment for multiple comparisons. The mean difference is significant at the 0.05 level.
